# Characterising functional strategies and trait space of freshwater macroinvertebrates

**DOI:** 10.1038/s41598-022-16472-0

**Published:** 2022-07-19

**Authors:** Dénes Schmera, Jani Heino, János Podani

**Affiliations:** 1 Balaton Limnological Research Institute, Klebelsberg K. u. 3, Tihany, 8237 Hungary; 2 Geography Research Unit, University of Oulu, P.O. Box 8000, 90014 Oulu, Finland; 3 Department of Plant Systematics, Ecology and Theoretical Biology, Institute of Biology, Eötvös Loránd University, Pázmány P. s. 1/C, Budapest, 1117 Hungary; 4 Institute of Evolution, Centre for Ecological Research, Budapest, Hungary

**Keywords:** Ecology, Community ecology, Freshwater ecology

## Abstract

We examined the functional strategies and the trait space of 596 European taxa of freshwater macroinvertebrates characterized by 63 fuzzy coded traits belonging to 11 trait groups. Principal component analysis was used to reduce trait dimensionality, to explain ecological strategies, and to quantify the trait space occupied by taxa. Null models were used to compare observed occupancy with theoretical models, and randomization-based analyses were performed to test whether taxonomic relatedness, a proxy of phylogenetic signal, constrains the functional trait space of freshwater macroinvertebrates. We identified four major strategies along which functional traits of the taxa examined show trade-offs. In agreement with expectations and in contrast to existing evidence we found that life cycles and aquatic strategies are important in shaping functional structure of freshwater macroinvertebrates. Our results showed that the taxonomic groups examined fill remarkably different niches in the functional trait space. We found that the functional trait space of freshwater macroinvertebrates is reduced compared to the range of possibilities that would exist if traits varied independently. The observed decrease was between 23.44 and 44.61% depending on the formulation of the null expectations. We demonstrated also that taxonomic relatedness constrains the functional trait space of macroinvertebrates.

## Introduction

Although the Earth is home to an astonishing level of biodiversity regarding forms, functions, and life histories of organisms, only a comparatively few essential trait combinations have been found to be common, widespread, and evolutionarily viable at present^1,2^. For example, the forms and functions of vascular plant species^1^ and invertebrates in tank bromeliads^2^ are strongly concentrated in the functional trait space. These findings call attention to general ecological strategies, which are defined as the combinations of key traits and can be considered as proxies for functional niche dimensions^3^.

Diaz et al.^4^ defined functional traits as follows: “Functional traits are morphological, biochemical, physiological, structural, phenological, or behavioural characteristics that are expressed in phenotypes of individual organisms and are considered relevant to the response of such organisms to the environment and/or their effects on ecosystem properties”. The functional trait space covered by a species assemblage is restricted by trade-offs among traits, as well as phylogenetic and ecological constraints^2^. First, organisms cannot optimize their performance in all niche dimensions simultaneously^5^. For instance, food gathering of stream macroinvertebrates is limited to the specific particle range size of their food, varying from very small (0.5 µm to 1 mm) for species feeding on fine organic material to relatively large (up to 10 cm) for those species preying on other organisms^6^. Second, past evolutionary constraints might also have influenced present-day patterns. For instance, based on studies of lizard communities, functional traits are assumed to be conserved at genus and family levels^7^. However, there is no clear evidence whether and how these findings apply to other organismal groups living in environments with different ecological constraints and selective pressures.

Freshwater macroinvertebrates (i.e., invertebrate animals longer than 0.25 mm^8^) consume various sources (e.g., algae, detritus, and other animals^6^), provide food for higher trophic levels^9^ and are therefore important components of riverine and lacustrine food webs^10^. Macroinvertebrates include a wide variety of taxonomic groups, ranging from sponges through annelids, molluscs, crustaceans to insects, each with distinct evolutionary histories. These animals inhabit a broad range of freshwater habitats, from small springs and temporary pools to large lakes and rivers. Obviously, a single species cannot exist in such a wide variety of habitats. It is indeed well-known that macroinvertebrates are strongly adapted to the habitat template^11,12^ through their response traits^13,14^ including also the direct and indirect effects of predatory fishes^15,16^, but less attention has been paid to evaluate how the effect traits^13^ are constrained and associated with ecological strategies. In their seminal paper, Usseglio-Polatera et al.^17^ showed that the overall structure of European freshwater macroinvertebrates is explained by traits related to reproduction, size and feeding habits, as well as that different taxonomic groups of macroinvertebrates represent relatively distinct strategies. This suggests that the strategies are, at least to some extent, evolutionarily conserved^18^. In addition, the traits of macroinvertebrates differ between temperate and Mediterranean regions^19^, and the trait profiles of invasive macroinvertebrates differ from those of native ones^20^. However, there has been a knowledge gap on how freshwater macroinvertebrates, in general, occupy the functional trait space within a specific regional species pool.

Here, we examine the ecological strategies and the functional trait space of European freshwater macroinvertebrates as exemplary cases. Relying on a functional trait database^21^, we first identify the functional traits primarily responsible for trait variation among macroinvertebrates. As previous studies relied on both effect and response traits and identified the importance of traits related to reproduction, size and feeding habits, we put forward the hypothesis that these traits define the major axes of variation in the functional trait space. Then, we determine the proportion of the functional trait space filled by macroinvertebrates. We predict that freshwater macroinvertebrates occupy a small part of the potential functional trait space due to trade-offs^22^ and evolutionary constrains^18^ between traits. This prediction agrees with other studies showing that vascular plants occupy 2% to 82% of the potential trait space^1^, while invertebrates living in tank bromeliads inhabit 16% to 23%^2^. Finally, we examined whether the occupancy of functional trait spaces is constrained by taxonomic relatedness which is considered as a proxy for phylogenetic signal. As existing evidence suggests that traits are relatively conserved at family level^23^, we predict that the concentration of taxa in the functional trait space is determined by taxonomic relatedness.

## Results

### Functional trait space of European macroinvertebrates

Based on the broken-stick distribution, the first 9 PCA axes were significant (Suppl. Table 1). The first four axes were correlated with multiple traits suggesting constraints shaping ecological strategies, while the next four PCA axes (5 to 8) were correlated only by a single trait each (Suppl. Table 2). We therefore interpreted the main axes of trait variation along the first four PCA axes, which revealed four strategies as detailed below. The first four axes of PCA explained individually more than 7% variance in the data, accounting for a total of 46.7%.

**Figure 1:**
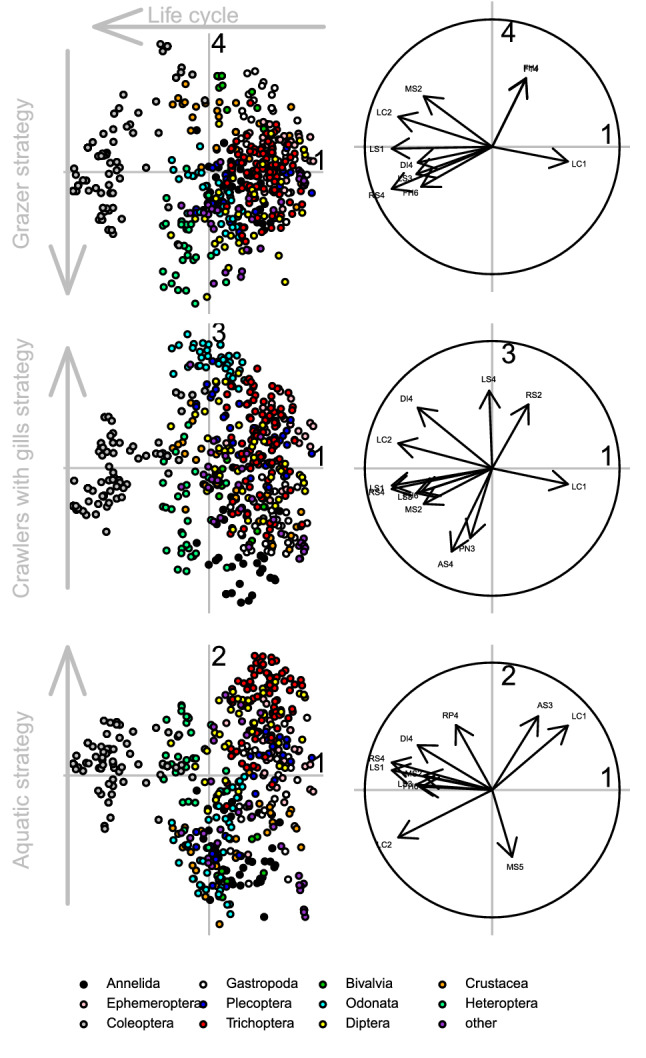
Centred Principal Component Analysis (PCA) ordination of freshwater macroinvertebrate taxa (left) according to their functional traits (right). The first four PCA axes (indicated by numbers) are depicted in three pairwise combinations (axis 1 versus axis 2, 3 or 4) and only functional traits with correlation r >|0.5| with at least one axis are shown. Grey arrows are used to interpret ecological strategies along the axes. See Table 4, for abbreviations of traits.

The first PCA axis (15.4%) represents life cycle (Fig. 1). It corresponds to a gradient from a short life cycle (≤ 1 year, LC1) at positive axis values to long life cycle (> 1 year, LC2) at negative axis values. The latter endpoint of the gradient can also be characterized by diverse strategies such as the piercer feeding habit (FH6), fliers (LS1), full water swimmers (LS3), respiration with spiracle (RS4), aerial active dispersal mode (DI4) and small size (MS2). The short life cycle is represented by mayflies (Ephemeroptera) and stoneflies (Plecoptera), while the long-lasting life cycle is shown by beetles (Coleoptera) and mussels (Bivalvia) (Fig. 1). The second PCA axis (9.5%) represents an aquatic strategy (Fig. 1). At the positive axis values, we identified the nymph aquatic stage (AS3) combined with reproduction with egg clutches, which are cemented or fixed (RP4). Several caddisfly (Trichoptera) taxa follow this strategy. The third PCA axis (9.2%) represents the crawlers with gills strategy (Fig. 1). We found the adult aquatic stage (AS4) and polyvoltine life cycle (PN3) at negative axis values while respiration with gills (RS2) and crawler locomotion (LS4) on the positive side. Negative scores are taken by worms (Annelida: Oligochaeta) and some mussels (Bivalvia), while dragonflies (Odonata) appear on the positive side. It seems that this is a unique and important strategy, where crawler locomotion is combined by with gills. The fourth PCA axis (7.1%) corresponds to the grazer strategy (Fig. 1). Negative axis values are correlated with the scraper habit (FH4) feeding on “microphytes” food type (FT4). Grazers feed on periphyton and are represented by multiple taxonomic groups. Some axes do not necessarily represent a gradient of strategies with well definable endpoints, but represent a single strategy, which causes substantial variation in the entire data set. Our analyses showed that mayflies (Ephemeroptera), beetles (Coleoptera), mussels (Bivalvia), caddisflies (Trichoptera), worms (Annelida), dragonflies (Odonata) and true bugs (Heteroptera) are positioned far from the origin of the PCA (Table 1), thereby contributing to functional niche space of the whole macroinvertebrate assemblage. Considering within-group heterogeneity, crustaceans (Crustacea), mayflies (Plecoptera), caddisflies (Trichoptera) and beetles (Coleoptera) showed the highest variance along the first four PCA axes (Table 2).

**Table 1:** The average position of taxonomic groups along the first four PCA axes.

Taxonomic group	Axis 1	Axis 2	Axis 3	Axis 4
Annelida	0.38	− 0.94	− **0.91**	− 0.20
Gastropoda	0.78	− 0.10	− 0.67	0.44
Bivalvia	0.28	− **1.14**	− 0.32	**1.01**
Crustacea	0.29	− 0.79	− 0.25	0.56
Ephemeroptera	**0.89**	0.24	0.38	0.23
Plecoptera	0.51	− 0.21	0.50	0.50
Odonata	0.07	− 0.81	**1.33**	− 0.34
Heteroptera	− 0.30	0.21	− 0.58	− **0.89**
Coleoptera	− **1.20**	0.15	− 0.01	0.20
Trichoptera	0.69	**0.80**	0.25	− 0.03
Diptera	0.50	0.51	0.11	− 0.46
Other (including several groups)	0.58	− 0.64	− 0.44	− 0.46

Minimum and maximum values are highlighted in bold.

**Table 2:** The variance of taxonomic groups along the first four PCA axes.

Taxonomic group	Axis 1	Axis 2	Axis 3	Axis 4
Annelida	0.08	0.12	0.26	0.10
Gastropoda	0.06	0.22	0.08	0.21
Bivalvia	0.05	0.05	0.09	0.05
Crustacea	**0.27**	0.13	0.26	0.14
Ephemeroptera	0.06	0.11	0.17	0.07
Plecoptera	0.17	**0.33**	0.15	0.09
Odonata	0.06	0.22	0.03	0.08
Heteroptera	0.05	0.19	0.27	0.18
Coleoptera	0.21	0.07	0.18	**0.48**
Trichoptera	0.05	0.18	**0.30**	0.10
Diptera	0.19	0.17	0.23	0.26
Other (including several groups)	0.30	0.64	0.33	0.17

Highest values are highlighted in bold (excluding the “other” group).

### Trait space occupied

The amount of functional trait space occupied by the whole macroinvertebrate assemblage was 18.92 (four-dimensional volume of the functional trait space assessed by the convex hull method). The volumes taken by taxonomic groups (Table 3) ranged from close to zero (mussels: Bivalvia) to 1.03 (true flies: Diptera). These values were lower than the expected proportional value 1.58 (= 18.92/12) suggesting that some or all individual taxonomic groups were functionally distinct from one another. We observed that insect orders of true flies (Diptera), beetles (Coleoptera) and caddisflies (Trichoptera) occupy the largest functional trait space among macroinvertebrate groups (Table 3). If the volume of the functional trait space was standardized by the number of taxonomic units in the group, then true flies (Diptera), true bugs (Heteroptera) and crustaceans (Crustacea) occupied the largest standardized functional space (Table 3).

**Table 3:** The amount and standardized amount of functional trait space occupied by the entire set of macroinvertebrates as well as by different taxonomic groups.

Organism group	Functional trait space	Standardized functional space (%)
Whole macroinvertebrate assemblage	18.92	3.17
Annelida	0.44	1.05
Gastropoda	0.48	1.17
Bivalvia	< 0.01	< 0.01
Crustacea	0.37	1.32
Ephemeroptera	0.25	0.68
Plecoptera	0.22	0.71
Odonata	0.13	0.33
Heteroptera	0.47	1.62
Coleoptera	0.85	0.49
Trichoptera	0.81	0.84
Diptera	1.03	2.71
Other (including several groups)	0.75	2.34

Standardized amount of functional trait space expresses the amount of functional trait space per taxon regarding the particular taxonomic group.

### Null models

The outlier-free amount of functional trait space of the macroinvertebrate community was 17.03. The realized hypervolume was only 23.44% (Model 1, uniform distribution), 35.29% (Model 2, normal distribution), and 44.61% (Model 3, random permutations) of the hypervolume predicted under the null hypotheses (*P* < 0.001 in all models). This means that the occupied functional trait space is strongly concentrated.

### Role of taxonomic relatedness

PERMANOVA showed that there were no significant differences in the functional distances among taxonomic groups (PERMANOVA, df = 11, R^2^ = 0.83, *P* = 0.509). However, if the “Others” group was broken into taxonomic groups, the functional distances were significantly different among taxa (PERMANOVA, df = 23, R^2^ = 0.99, *P* = 0.001). We observed similar result when the “Others” group was omitted from the analyses (PERMANOVA, df = 10, R^2^ = 0.99, *P* = 0.001). PERMDISP showed that the within-group variation differed among taxonomic groups with the aggregation of “Others” (PERMDISP, F = 6.926, *P* < 0.001), without the aggregation of “Others” (PERMDISP, F = 6.558, *P* < 0.001), as well as when “Others” were omitted (PERMDISP, F = 304, *P* < 0.001). In general, the largest variation in within-group distances was found in beetles (average distance to the median: 0.873), flies (0.870) and crustaceans (0.823), and the lowest variation in within-group distances was detected in small groups (Brachiobdellea, Hymenoptera, Nemertea and Polychaeta, average distance to the median equals to 0).

## Discussion

We identified four major strategies along which functional traits of freshwater macroinvertebrates show trade-offs. We found that the major taxonomic groups examined here fill different niches in the functional trait space. We observed that the occupied functional trait space was restricted compared to the range of possibilities that would exist if traits varied independently. We demonstrated also that taxonomic relatedness, a proxy for possible phylogenetic signal, constrains the functional trait space of freshwater macroinvertebrates. All these findings suggest that the analysis of the functional trait space of freshwater macroinvertebrates allows identifying functional differences among different groups, elucidates constraints of evolution, and may contribute to better understanding of functional features of ecological communities.

We identified the life cycle, aquatic, crawlers with gills and grazer strategies, along which the traits of freshwater macroinvertebrates showed trade-offs. This finding partly agrees with the study of Usseglio-Polatera et al.^17^, who emphasized the importance of size, reproduction and feeding habits in structuring freshwater macroinvertebrate assemblages. The grazing strategy defined by us can correspond to the feeding habit strategy defined in^17^. We found that different taxonomic groups fill different niches in the functional trait space. This observation agrees with the results of Usseglio-Polatera et al.^17^ who suggest that major taxonomic groups follow different and well-separable ecological strategies. There are, however, several disagreements between the findings of the two studies. Most importantly, Usseglio-Polatera et al. (^17^, p. 190) concluded that “surprisingly, two a priori ‘important’ life cycle attributes (i.e. life duration and aquatic stages of eggs, larvae, pupae, or adults) contributed poorly to the biological typology of macroinvertebrates”. Our results, however, do indicate that life cycle and aquatic strategies are influential traits in shaping the functional variation among freshwater macroinvertebrates.

A novel aspect of our study is that while Usseglio-Polatera et al.^17^ identified gradients of different strategies, our results suggest that contrasting strategies cannot necessarily be envisioned along gradients of the functional trait space, although axis 1 in our PCA results was found to be associated with the life cycle. It seems that certain unique strategies of freshwater macroinvertebrates do have more substantial contribution to variation in the functional trait space than others, being restricted to a well-defined and distinct part of that space. The PCA results clearly suggested such strategies, showing that the PCA axes could not be interpreted as a gradient of strategies with two easily definable endpoints.

In agreement with the relevant literature^17^, PCA analyses revealed that the taxonomic groups fill different niches in the functional trait space. We found that mayflies (Ephemeroptera), beetles (Coleoptera), mussels (Bivalvia) and caddisflies (Trichoptera) are far from the origin in the first two ordination dimensions and thus greatly contribute to the occupancy of the functional niche space. The results of Usseglio-Polatera et al. (^17^, see their Fig. 2) suggest, however, that smaller groups (Bryozoa, Porifera, Lepidoptera) and true bugs (Heteroptera) have strong contribution. We found that crustaceans (Crustacea), stoneflies (Plecoptera), caddisflies (Trichoptera) and beetles (Coleoptera) cause the largest variance along ordination axes. The results of Usseglio-Polatera et al. (^17^, see their Fig. 2) show the high variance of stoneflies (Plecoptera), beetles (Coleoptera) and caddisflies (Trichoptera).

Obviously, there are some differences between the methodology of Usseglio-Polatera et al.^17^ and that of the present study. These differences might explain some disagreements. First, Usseglio-Polatera et al.^17^ examined small groups (Bryozoa, Porifera, Cnidaria, and some others) separately while we aggregated them into a single group (“Others”). Second, Usseglio-Polatera et al. (^17^, p. 177) coded missing data by zero affinity score and stated that “This ensured that in multivariate analyses this 'not documented' taxon was treated with the average profile of all other taxa for the corresponding variable; in other words, its discriminative weight for this particular variable was zero (Chevenet et al. 1994)”. We argue that replacing missing data by zero scores indicating no affinity is not an ideal solution because zero is not the average of all other taxa in most cases, or if it is, then the trait has no discriminative power and thus must be omitted from further analyses. We, in contrast, did not replace missing data values by zero, and used a novel approach to PCA that can handle missing data^24^. Third, Usseglio-Polatera et al.^17^ used raw fuzzy coded traits, while the present study used standardized trait scores. Our standardization allowed to handle weight differences and relatedness of traits. Fourth, Usseglio-Polatera et al.^17^ used fuzzy correspondence analysis (CA), whereas the present study applied Principal Component Analysis (PCA). While fuzzy CA is a highly sophisticated ordination method in which the term “correspondence” refers to the mutual position of objects and variables in their joint plot, PCA is a basic procedure of multivariate data exploration, where the ordination of objects and variables are obtained separately and superimposed over one another afterwards to form a biplot^25^. We selected PCA because recently developed approaches to the study of trait space occupation rely on PCA^1,2^. Finally, Usseglio-Polatera et al.^17^ examined two axes (explaining 17.9% of total variability), whereas we examined four axes (accounting for 47.7% of total variability). These methodological differences may explain disagreements between the findings of Usseglio-Polatera et al.^17^ and the present study.

Ours is the first study that examines the trait space occupancy of freshwater macroinvertebrates along several dimensions simultaneously. Although Usseglio-Polatera et al.^17^ already examined the distribution of macroinvertebrates along different ordination axes separately, the use of convex hulls allows to obtain a wider view of trait variation. According to our results, true flies (Diptera), true bugs (Heteroptera) and crustaceans (Crustacea) occupy the largest standardized functional trait space. These findings are novel, because previous analyses based on individual axes highlighted the importance of other taxonomic groups (^17^, present study). At the same time, the emergence of these three taxa agrees with expectations, because true flies, true bugs and crustaceans potentially show considerable variability in ecosystem functioning^26^.

We found that the functional trait space of freshwater macroinvertebrates is reduced compared to the range of possibilities that would exist if traits varied independently. This finding arises from the analyses of 63 fuzzy coded traits^17,27^ describing feeding, locomotion, food, respiration, size, resistance, dispersal, aquatic stage, life cycle duration, number of cycles and reproduction of macroinvertebrates. The observed reduction of trait space varied between 23.44 and 44.61%, depending on the formulation of the null expectations. These values have a narrower range than observed in plant communities (2–82%^1^), potentially indicating much higher variation of functionality in the terrestrial environment or for plants in general. A possible explanation for such values is that plants have much higher phenotypic plasticity than animals. However, macroinvertebrates in lakes and rivers are functionally less similar compared with those living in the very special aquatic habitat of tank bromeliads (16.29% and 23.35%^2^). We found evidence that this restriction is constrained by ecological features and taxonomy as a proxy of phylogenetic relatedness.

The observed patterns and identified mechanisms have several consequences. From a theoretical point of view, we can conclude that individual traits of freshwater macroinvertebrate taxa are inter-dependent due to phylogenetic and ecological constraints. Considerable inter-dependence of traits and the consequent reduction of trait space occupancy cannot be ignored in understanding and enhancing biodiversity restoration and community functionality. For instance, attempts to enhance functional diversity of freshwater macroinvertebrates through habitat restoration cannot focus exclusively on the traits related to the specific ecosystem function required (e.g., detritus processing), but should also consider how such traits are linked to other traits and constrained through the ecology and evolution of different species. We argue that the analytical approach used here promotes understanding functional aspects of assemblages in light of evolutionary constraints. This approach can be applied widely in biogeography and ecology, as well as applied studies focusing on understanding the functional facets of biodiversity.

## Methods

### Functional traits and their use in the present study

A trait database for European freshwater macroinvertebrates^26^ was downloaded from the *freshwaterecology.info* website^21,28^. Following the terminology of Schmera et al.^29^, this database contains fuzzy coded traits^27^ grouped into trait groups. Traits were scored by experts (see^30^). This means that experts quantified the relative importance of a particular trait for a taxon or, in other words, the relative affinity of a taxon to a particular trait (e.g., is the 'absorber' feeding mode characteristic to a given taxon?). Depending on the number of traits within a particular trait group, trait states were coded by an integer score ranging from 0 (no affinity) to 3 (high affinity) [for trait groups with low number of traits], or by an integer score ranging from 0 (no affinity) to 5 (high affinity) [for trait groups with high number of traits]. Although not stated explicitly, fuzzy coding^27^ uses ratio (rather than ordinal) scale because both the original developers of the coding system and the first applications^17^ clearly stated that scores are to be converted into % trait^29^.

To make the data set more representative of the entire European continent, we added 44 taxa from the Mediterranean area^31^, thus increasing the number of taxa to 596. The Mediterranean data set had only 4 traits instead of 5 in the trait group of respiration (by ignoring the hydrostatic vesicle trait, RS5 in Table 4) and it lacked the absorber trait from the feeding habit trait group (FH1 in Table 4). In both cases, 0 s were added to these traits assuming that taxon affinities for these traits were zero^30^. The trait “Detritus < 1 mm” from the Mediterranean set was matched to the “Fine detritus (≤ 1 mm)” trait in the Usseglio-Polatera data set and “Plant detritus ≥ 1 mm” was matched to “Dead plant (> 1 mm)”. Finally, our data set consisted of 11 trait groups with a total of 63 traits (Table 4). The taxonomic resolution in the data ranges from the species through genus, tribe, subfamily, and family levels to orders. Considering the *freshwaterecology.info* website^21,28^ and taking into account the Fauna Europaea database^32^, we assigned the taxa to 12 major groups. These groups are widely used taxonomic groups in macroinvertebrate research with several records (> 10) in our database (Annelida, Gastropoda, Bivalvia, Crustacea, Ephemeroptera, Plecoptera, Odonata, Heteroptera, Coleoptera, Trichoptera, and Diptera). One group (called here “Others”) represents further taxonomic groups, each with limited number of records (< 10) in our database (Porifera, Cnidaria, Bryozoa, Platyhelminthes, Nemertea, Nematomorpha, Hymenoptera, Lepidoptera, Megaloptera, and Neuroptera). Although this group is visualized as "Others", it is broken into real taxonomic groups in the statistical analyses. As the number of taxa within taxonomic groups varied considerably due to the low number of taxa in the “Others” group and because the results might be sensitive to differences in the number of taxa, we run analyses also without the "Others".

**Table 4:** List of functional trait groups and functional traits used in our analyses.

Trait group	Trait	Abbreviations
Feeding habit	Absorber	FH1
	Deposit feeder	FH2
	Shredder	FH3
	Scraper	FH4
	Filter-feeder	FH5
	Piercer (plants or animals)	FH6
	Predator (carver/engulfer/swallower)	FH7
	Parasite	FH8
Locomotion and substrate relation	Flier	LS1
	Surface swimmer	LS2
	Full water swimmer	LS3
	Crawler	LS4
	Burrower (epibenthic)	LS5
	Interstitial (endobenthic)	LS6
	Temporarily attached	LS7
	Permanently attached	LS8
Food type	Fine sediment + microorganisms	FT1
	Fine detritus (≤ 1 mm)	FT2
	Dead plant (> 1 mm)	FT3
	Living microphytes	FT4
	Living macrophytes	FT5
	Dead animal (> 1 mm)	FT6
	Living microinvertebrates	FT7
	Living macroinvertebrates	FT8
	Vertebrates	FT9
Respiration	Tegument (respiration through the body surface)	RS1
	Gill (respiration using special respiration organs)	RS2
	Plastron (respiration using a thin layer of air around the body)	RS3
	Spiracle (aerial) (respiration using small openings on the body surface)	RS4
	Hydrostatic vesicle (aerial) (respiration using air within a small blister)	RS5
Maximal potential size	≤ 0.25 cm	MS1
	> 0.25–0.5 cm	MS2
	> 0.5–1 cm	MS3
	> 1–2 cm	MS4
	> 2–4 cm	MS5
	> 4–8 cm	MS6
	> 8 cm	MS7
Resistance form	Eggs, gemmula, statoblasts	RF1
	Cocoon	RF2
	Housing against desiccation	RF3
	Diapause or dormancy	RF4
	None	RF5
Dispersal	Aquatic passive	DI1
	Aquatic active	DI2
	Aerial passive	DI3
	Aerial active	DI4
Aquatic stage	Egg	AS1
	Larva	AS2
	Nymph	AS3
	Adult	AS4
Life cycle duration period	≤ 1 year	LC1
	> 1 year	LC2
Potential number of cycles per year	< 1, semivoltine	PN1
	1 monovoltine	PN2
	> 1 polyvoltine	PN3
Reproduction	Ovoviviparity	RP1
	Isolated eggs, free	RP2
	Isolated eggs, cemented	RP3
	Clutches, cemented or fixed	RP4
	Clutches, free	RP5
	Clutches, in vegetation	RP6
	Clutches, terrestrial	RP7
	Asexual reproduction	RP8

### Data analysis

In our database, trait groups include a set of related traits^29^. This means that the score of the “absorber” trait, for instance, should be assessed and interpreted in relation to the other trait scores within the same trait group (here: “Feeding habit” trait group, see Table 4). Consequently, the standardization of each trait separately, which is a common practice for independent traits^1^, would destroy the data structure of related traits. Moreover, the range difference of affinity scores between trait groups with low (range: 0 to 3) and high (range: 0 to 5) number of traits may assign different and unintended weights to the traits^33^. To address both issues, we standardized trait scores *of each trait group separately* to the interval [0,1]. In this way, we obtained a standardized taxa-by-traits data matrix, which maintained the relatedness of traits. A relatively low number of entries (388, 1.03%) in the taxa-by-traits data matrix were unknown or missing.

Centred Principal Component Analysis (centred PCA^25^) of incomplete data^24^ was used to produce the trait space of European freshwater macroinvertebrates. We deliberately avoided the use of standardized PCA, in which traits are normalized to zero mean and unit variance, because such data transformation would destroy the existing relatedness of traits. The number of significant axes of the PCA ordination was determined based on the broken-stick distribution model^34^. The correlations between traits and ordination axes were used to assess the contribution of traits to the ordination as suggested by Legendre & Legendre^34^. Following Céréghino et al.^2^, we retained traits with a correlation r >|0.5| with a given axis.

To assess the functional trait space occupied by macroinvertebrate taxa, we calculated the multidimensional convex hull volume following Cornwell et al.^35^. This functional trait space was compared to three null models following Céréghino et al.^2^. The null models represent the hypothesis that the scores on the selected ordination axes follow a standard statistical distribution. Model 1 assumes that simulated component scores are uniformly distributed in the trait space, Model 2 assumes that simulated component scores are normally distributed, while Model 3 assumes that the observed component scores are randomly and independently permuted on each axis. To control for outliers, convex hull volumes were computed on the observed and simulated convex hulls containing 95% of taxa located closest to the centroid^1^.

The phylogenetic signal cannot be directly tested because the phylogeny of European macroinvertebrates is still incomplete. We therefore tested the concentration of related taxa in the functional trait space by comparing within and between group trait-dissimilarities. We used the component scores of each taxon in permutational analysis of variance (PERMANOVA, Euclidean distance, 999 permutations^36^) to test whether taxa are significantly more dissimilar between groups than within groups. Thus, PERMANOVA shows whether different taxonomic groups occupy different parts of the trait space. We also examined the homogeneity of multivariate dispersion (PERMDISP, Euclidean distance, 999 permutations^37^). PERMDISP tests whether the within-group trait variation of the studied groups differs. In ecological terms, this would indicate which taxonomic groups show most (or least) among-taxon variation in the functional traits analysed and, thus, in heterogeneity (or homogeneity) along the axes of ecological strategies. All analyses were performed in the *R* environment^38^ using the *BiodiversityR*^39^, *geometry*^40^ and *vegan*^41^ packages. Centred PCA of incomplete data was performed also in the R environment^38^ using program InDaPCA^24^.

## Supplementary Information

Supplementary Tables.

## Data Availability

The datasets generated and/or analysed during the current study are available in freshwaterecology.info website^21,28^.
