# Supplementary material for: Characterising functional strategies and trait space of freshwater macroinvertebrates
Source: Sci Rep. 2022 Jul 19;12:12283. doi: 10.1038/s41598-022-16472-0 (PMC9296484; doi:10.1038/s41598-022-16472-0)
Supplement: Supplementary file 1 — Supplementary Tables. [file 41598_2022_16472_MOESM1_ESM.docx]

**Supplementary documents**

Suppl. Table 1. The percentage of variance explained by PCA and by the broken-stick model

|  | PCA axes | | | | | | | | | |
| --- | --- | --- | --- | --- | --- | --- | --- | --- | --- | --- |
|  | 1 | 2 | 3 | 4 | 5 | 6 | 7 | 8 | 9 | 10 |
| percentage of variance explained by PCA | 15.4 | 9.4 | 9.2 | 7.1 | 5.7 | 5.1 | 3.8 | 3.6 | 3.6 | 3.0 |
| percentage of variance based on broken-stick model | 7.5 | 5.9 | 5.1 | 4.6 | 4.2 | 3.9 | 3.6 | 3.4 | 3.2 | 3.0 |

Suppl. Table 2. Traits showing high correlation (r > |0.5|) with a given axis. Trait codes are given in Table 1 of the main document. NT means that there is no trait with high correlation.

| Axis | Trait | Correlation |
| --- | --- | --- |
| 1 | FH6 | -0.556 |
|  | LS1 | -0.788 |
|  | LS3 | -0.595 |
|  | RS4 | -0.788 |
|  | MS2 | -0.537 |
|  | DI4 | -0.584 |
|  | LC1 | 0.594 |
|  | LC2 | -0.737 |
| 2 | MS5 | -0.522 |
|  | AS3 | 0.579 |
|  | LC1 | 0.505 |
|  | RP4 | 0.509 |
| 3 | LS4 | 0.609 |
|  | RS2 | 0.502 |
|  | AS4 | -0.553 |
|  | PN3 | -0.543 |
| 4 | FT4 | 0.531 |
|  | FH4 | 0.539 |
| 5 | RS1 | -0.598 |
| 6 | PN2 | -0.634 |
| 7 | MS3 | -0.555 |
| 8 | MS4 | 0.606 |
| 9 | NT |  |
| 10 | NT |  |
